# Combining Drought Survival via Summer Dormancy and Annual Biomass Productivity in *Dactylis glomerata* L.

**DOI:** 10.3389/fpls.2016.00082

**Published:** 2016-02-09

**Authors:** Rajae Kallida, Latifa Zhouri, Florence Volaire, Adrien Guerin, Bernadette Julier, Naima Shaimi, Malika Fakiri, Philippe Barre

**Affiliations:** ^1^Unité de Recherche de Production Animales et Fourrage, INRA Maroc, Centre Régional de la Recherche Agronomique de RabatRabat, Morocco; ^2^Laboratoire d’agroalimentaire et santé, Faculté des Sciences Techniques de Settat, Université Hassan 1erSettat, Morocco; ^3^USC 1338, Centre d’Ecologie Fonctionnelle et Evolutive, UMR 5175, Institut National de la Recherche AgronomiqueMontpellier, France; ^4^UR4 Unité de Recherche Pluridisciplinaire Prairies et Plantes Fourragères, Institut National de la Recherche AgronomiqueLusignan, France; ^5^Unité de Recherche d’Amélioration des Plantes Valorisation et Conservation des Ressources Phytogénétiques, INRA Maroc, Centre Régional de la Recherche Agronomique de RabatRabat, Morocco

**Keywords:** forage, cocksfoot, orchard grass, drought stress, QTL, auto-tetraploid

## Abstract

Under Mediterranean climates, the best strategy to produce rain-fed fodder crops is to develop perennial drought resistant varieties. Summer dormancy present in native germplasm has been shown to confer a high level of survival under severe drought. Nevertheless it has also been shown to be negatively correlated with annual biomass productivity. The aim of this study was to analyze the correlations between summer dormancy and annual biomass productivity related traits and to identify quantitative trait loci (QTL) for these traits in a progeny of a summer dormant cocksfoot parent (Kasbah) and a summer active parent (Medly). A total of 283 offspring and the parents were phenotyped for summer dormancy, plant growth rate (PGR) and heading date in Morocco and for maximum leaf elongation rate (LERm) in France. The individuals were genotyped with a total of 325 markers including 59 AFLP, 64 SSR, and 202 DArT markers. The offspring exhibited a large quantitative variation for all measured traits. Summer dormancy showed a negative correlation with both PGR (-0.34 *p* < 0.005) and LERm (-0.27 *p* < 0.005). However, genotypes with both a high level of summer dormancy and a high level of PGR were detected in the progeny. One genetic map per parent was built with a total length of 377 and 423 cM for Kasbah and Medly, respectively. Both different and co-localized QTL for summer dormancy and PGR were identified. These results demonstrate that it should be possible to create summer dormant cocksfoot varieties with a high annual biomass productivity.

## Introduction

In the context of global climate change, the frequency and intensity of summer droughts are predicted to increase and therefore the areas under Mediterranean-like climates may extend (IPCC, 2014). In these harsher environments, the best strategy to produce rain-fed fodder crops is to create perennial drought resistant varieties suitable for large areas including semi-arid regions (Volaire et al., 2013; Malinowski and Pinchak, 2015).

Native germplasm of perennial grasses such as cocksfoot (*Dactylis glomerata* L.) and tall fescue (*Festuca arundinacea* subsp *arundinaceae* Schreb.), which are the most grown perennial forage species in semi-arid environments (Sleper and West, 1996; Reed et al., 1999; Nie et al., 2004, 2008), combine significant winter growth with high summer drought tolerance (Lelievre and Volaire, 2009). Recent studies confirmed that adapted material could be found both for tall fescue (Pecetti et al., 2011) and cocksfoot (Annicchiarico et al., 2011) within Mediterranean plant material evaluated across a set of drought-prone locations of the Mediterranean basin. Under the Mediterranean climate, perennial grasses can cope with drought and heat through different adaptive strategies. These go from resistance to moderate drought with growth maintenance to survival under severe stress with growth cessation (Volaire et al., 2014). In addition, since summer droughts are chronic in Mediterranean areas, some perennial herbaceous species exhibit a strategy of summer dormancy independent from stress (Volaire and Norton, 2006).

In some grass species, summer dormancy confers the endogenous ability to cease aerial growth and to senesce irrespectively of water supply in summer (Volaire and Norton, 2006). The environmental factors that trigger summer dormancy are not completely elucidated but summer dormancy is induced under increasing photoperiod and temperature conditions (Volaire and Norton, 2006). Under summer irrigation, summer dormancy can be either complete when leaf growth is stopped for a few weeks and most mature aerial biomass senesces, or incomplete when leaf growth is markedly reduced but without dehydration of aerial tissues (Volaire and Norton, 2006).

Summer dormancy is a valuable strategy associated with increased drought tolerance and persistence in perennial grasses (Hopkins and Bhamidimarri, 2009). It has been reported that summer dormancy, whether complete or incomplete, is associated with superior survival and autumn regrowth after severe and repeated summer drought (Volaire and Norton, 2006; Shaimi et al., 2009b), in cocksfoot (Norton et al., 2006a), tall fescue (Norton et al., 2006b), and phalaris (Culvenor and Boschma, 2005).

Nevertheless, complete summer dormancy has been shown to be associated with low annual productivity in natural populations (Norton et al., 2008; Shaimi et al., 2009a). It is not known if this negative correlation is due to the fact that the two traits are controlled by the same genes or highly linked genes leading to a physiological trade-off, or to the fact that both traits responded to natural selection in opposite directions. In the latter case, it should be possible to break this correlation and to create improved plant material combining acceptable levels of summer dormancy and high productivity in rainy seasons. A better knowledge in the genetic and eco-physiological determinisms of both summer dormancy and annual productivity is needed to evaluate the possibility of genetic progress for adaptation of perennial herbaceous forage species to semi-arid areas.

*Dactylis glomerata* L. is an auto-tetraploid species (4x = 28) for which molecular genetic studies are laborious (Hackett et al., 1998). Some simple sequence repeat (SSR) markers have been developed (Bushman et al., 2011; Hirata et al., 2011) but the genomic sequence is not available. Very few genetic maps (Song et al., 2011; Xie et al., 2012b) and only one quantitative trait loci (QTL) study on heading date (HD; Xie et al., 2012a) have been published.

The objectives of this research are for the first time (i) to analyze the genetic correlations between summer dormancy and biomass productivity in spring and autumn and (ii) to identify QTL for these traits through the analysis of a progeny obtained by crossing a summer dormant cocksfoot exhibiting low annual biomass productivity and a productive summer active cocksfoot. This study was carried out in two sites with contrasting environments: the Mediterranean and Oceanic climates respectively.

## Materials and Methods

### Plant Material

A cross between two genotypes of *D. glomerata* L. (cocksfoot), one from the summer dormant cultivar ‘Kasbah’ from Morocco and bred in Australia (Oram, 1990) and one from the summer active Mediterranean cultivar ‘Medly,’ was performed in 2009. Medly is of Mediterranean origin and was bred in the south of France (Volaire, 2002). Kasbah is less productive than Medly but more tolerant to severe drought (Annicchiarico et al., 2011; Lelievre et al., 2011). The two plants were cloned and grown in a field in Morocco and were well irrigated until their reproductive maturity. At heading stage, plants were covered by pollen-proof cages and crossings were completed without castration. A wood stick was used every day to shake the plants and thus enhance pollination. At maturity, seeds were harvested from both parents. About 300 seeds from each parent were germinated in Petri dishes and were transplanted into pots. The offspring status was checked on 285 plants by using amplified fragment length polymorphism (AFLP) markers (Vos et al., 1995), thus leading to discarding only two plants. Finally, the experiment was carried out with 283 offspring: 185 from Kasbah as a mother and 98 from Medly as a mother. All offspring and both parents were cloned to provide genetically identical plants for the experiments that were carried out both in the field in Rabat (Morocco) and in glasshouse in Lusignan (France). A plant from a temperate cultivar of cocksfoot (Ludac) was used as a non-summer dormant and non-Mediterranean control genotype. Ludac was chosen for its high biomass productivity in temperate and wet areas.

### Field Experiment in Morocco

Three clones of each of the 283 offspring, the two parents and Ludac were transplanted at the two tiller stage in the field on February 2011 at the Guich experimental station of INRA, Rabat/Morocco (34°03′N, 06°46′W). All offspring were transplanted as spaced plants with 100 cm between plants and rows. The experimental design was a completely randomized three block design. The soil was a mixed, thermic Lamellic Xeropsamments in the USDA classification (pH 6.8 and organic matter content 1.2%) without depth limitation for root growth and was fertilized with 28 kg/ha of N, 56 kg/ha of P, and 28 kg/ha of K before planting. Nitrogen was provided after each harvest (40 kg/ha of N). The plants were maintained weed free mainly by hand weeding and by covering the soil with a white plastic sheet around the plants. The trial was irrigated to maintain optimum water supply. A cut with a mower was performed in May 2011.

All measurements were made on each of the 858 plants. In 2012, after a hand cut (at a 5 cm height) performed on the 31st of January, stretched plant height was measured weekly during 6 weeks. These series of data on each plant were used to estimate the plant growth rate (PGR) during the linear phase of re-growth by fitting a linear curve between the plant height in mm and the thermal time (base temperature equal zero) in degree days (°Cd). PGR on spaced plant is correlated to leaf elongation rate which is correlated to forage yield in sward (Rhodes, 1969, 1971, 1973; Horst et al., 1978). HD was assessed every 2–3 days from end of February to beginning of April and was defined by the date when three spikes were visible (days from the first of January). HD is an important trait used to characterize cultivars in order to determine in which environmental conditions they are the most favorable. Summer dormancy (Dorm) was estimated by a scoring of herbage senescence (visual scoring with 0 = all tissues are green and 100 = no visible green tissues) at the beginning of the summer, on the 16th of July, under irrigation (Norton et al., 2008). Summer dormancy is correlated to severe drought resistance (Hopkins and Bhamidimarri, 2009).

### Glasshouse Experiment in France

Phenotyping of leaf growth was performed on three clones for each of the 283 offspring, the parents and Ludac according to Auzanneau et al. (2011), at Lusignan (46°24′16.1″N; 00°04′44.9″E), France. Three clones per genotype, obtained from the same plants as in Morocco, were installed on the 14th of September 2011 in trays with 15 cm of peat on a 2 cm layer of sand. The experimental design was a completely randomized three block design. After 3 weeks of growth, on the 10th of October, the plants were cut at a 5 cm height and leaf length measurements were carried out three times weekly on the third and fourth entire leaves after emergence from the 26th of October to the 12th of December. Maximum leaf elongation rate (LERm) was estimated by fitting leaf length in mm and thermal time in °Cd with a beta function. For each plant with a good fitting for both leaves, the average of LERm between the third and fourth entire leaves was used for further analysis. LERm has been proved to be the main factor affecting forage yield in sward (Rhodes, 1969, 1971; Horst et al., 1978; Reeder et al., 1984). Moreover, selection on LERm or leaf length measured on spaced plants modifies the forage yield in dense canopies (Rhodes and Mee, 1980; Hazard, 1996; Hazard and Ghesquière, 1997).

### Genotyping

For each genotype, i.e., the parents and their offspring, DNA was extracted from 50 mg of fresh leaves dried *in silica* gel with the the same protocol as in Pauly et al. (2012), i.e., CTAB (hexadecyltrimethylammonium bromide) lysis followed by chloroform octanol. The 283 offspring and the two parents were genotyped with a total of 325 markers, including 59 AFLP, 64 SSR, and 202 Diversity Array Technology (DArT) markers. The SSR primers were from Bushman et al. (2011). AFLP and SSR markers were obtained at INRA-URP3F, Lusignan, France, following the same protocol as described in Pauly et al. (2012). AFLP markers and SSR alleles were scored as presence/absence. DArT markers were obtained on the La Valette campus CIRAD platform of genotyping, under the supervision of Pierre Mournet (pierre.mournet@cirad.fr), following the same protocol as described in Phung et al. (2014). The library of cocksfoot DNA fragments was based on 10 varieties and 22 ecotypes with one individual per population (Ghesquière et al., 2012; Ghesquiére, 2013). Individuals, arranged in three 96 well plates were genotyped. The scoring was performed for each plate separately based on the TargetRatioMed corresponding to the ratio of fluorescence between the sample and a control. The Mclust R package http://cran.r-project.org/web/packages/mclust/index.html was used to cluster individuals into two groups. Individuals with a probability higher than 0.75 of belonging to one group were scored 1 if they belonged to the group with the highest values of TargetRatioMed or 0 if they belonged to the group with the lowest values of TargetRatioMed. All the other individuals were scored as missing data. If the number of missing data was higher than 20%, the marker was discarded. For each remaining marker, the three histograms of the TargetRatioMed corresponding to the three plates were drawn and checked manually for the presence of two distinct modes. Finally, based on replicated genotypes, markers with repeatability lower than 80% were discarded.

### Statistical Analysis

For each trait, genotypes with two missing data or outliers were omitted leading to 199 studied genotypes for LERm and 183 studied genotypes for PGR, HD, and Dorm. A variance analysis was performed on the offspring for each trait with block as fixed effect and genotype as random effect [PROC GLM of (SAS Institute, 2011)]. In this procedure, the adjusted least square means were calculated per genotype for each trait with LSMEANS. The error and genotype variances (σ^2^*_error_* and σ^2^*_geno_*, respectively) were estimated using the SAS Varcomp procedure. Broad sense heritability (H^2^) was calculated as H^2^ = σ^2^*_geno_*/(σ^2^*_geno_* + σ^2^*_error_*/3). The basic statistics (average, maximum, minimum, standard deviation, normality with the Shapiro–Wilk test, Pearson’s correlation) and graphics based on adjusted means were performed using Statistica (StatSoft, 2003).

The genetic maps were built with the genotypic data from the two parents and the offspring using the TetraploidMap software, following the manual guide instructions (Hackett and Luo, 2003). Only markers with a segregation not significantly different from 1:1 (simplex) or 1:5 (duplex) or 1:3 (double simplex) with *p* < 0.05 were included in the analysis. For the clustering step, each allele of multi-allelic markers was scored as presence/absence. Based on the clustering (dendogram), seven linkage groups (LGs) were established for each parent. LOD score (logarithm of the odds) ≥ 3.0, i.e., the probability that two loci are linked is 1000 times higher than the probability that they are not linked and recombination frequencies < 0.5 were used as thresholds to detect genetic linkage. Marker ordering within LGs was performed by using the four options proposed by TetraploidMap: 1/ two-point linkage analysis, 2/ initial-run ‘custom’ marker ordering, 3/ ripple ordering, and 4/ simulated annealing ordering. A consensus map has been built with the consensus map creation (ConsMap) analysis of the software BiomercatorV4 (Sosnowski et al., 2012).

Quantitative trait loci identification was performed by variance analysis (PROC GLM of SAS) for each marker. Markers with a significant effect (*p*-value < 0.01) were retained. For each parent, a multiple linear regression with the stepwise option (PROC REG of SAS) was performed with the significant markers in order to define the final markers considered as QTL and estimate the percentage of phenotypic variance explained by the QTL.

## Results

### Trait Heritability and Variability

**FIGURE 1:**
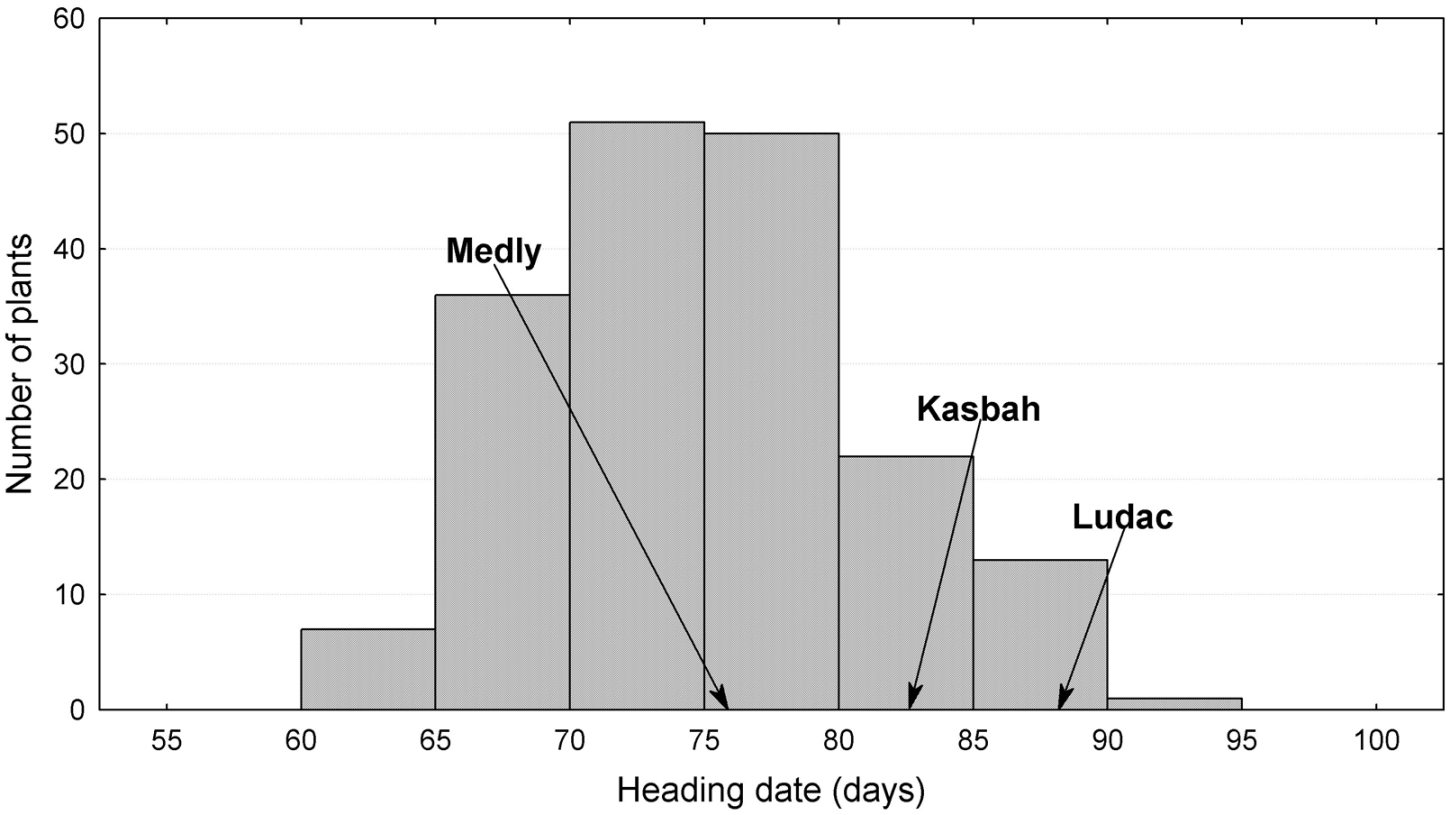
**Distribution of heading date (HD) for the offspring between two *Dactylis glomerata* L. individuals from the summer dormant cultivar Kasbah and the summer active cultivar Medly.** Ludac is indicated as a control for a temperate individual.

**FIGURE 2:**
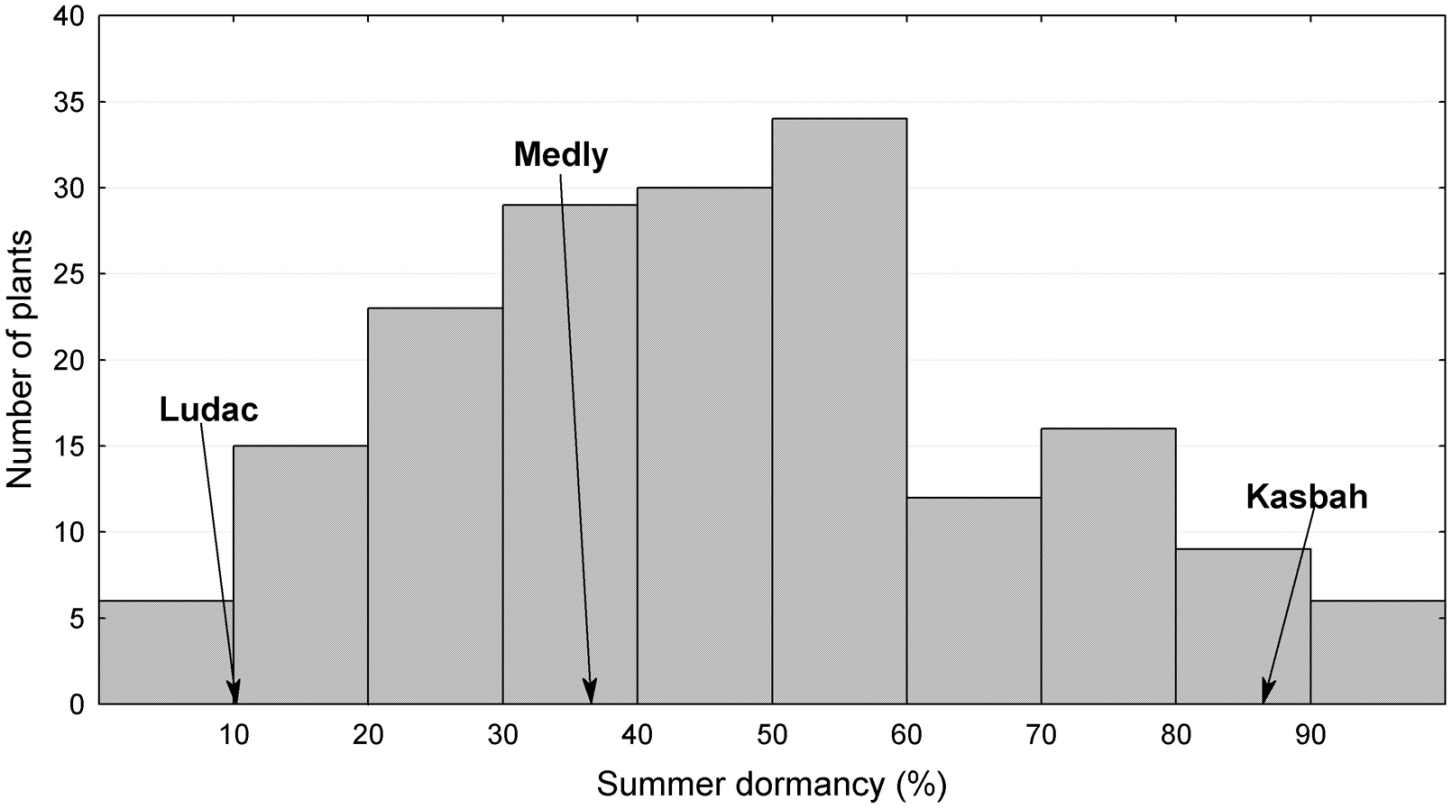
**Distribution of summer dormancy for the offspring between two *D. glomerata* L. individuals from the summer dormant cultivar Kasbah and the summer active cultivar Medly.** Ludac is indicated as a control for a temperate individual.

The broad sense heritability of the traits varied from 0.48 for PGR to 0.67 for HD (**Table 1**). All traits exhibited a large variability within the progeny and showed transgressive segregation compared to Kasbah and Medly (**Table 1**). As expected, Medly and Ludac showed a higher vegetative growth rate than Kasbah, both in the spring in the field in Morocco and in the autumn in the glasshouse in France. As expected, Kasbah exhibited a much higher level of summer dormancy than Medly and Ludac. Medly flowered earlier than Kasbah and Ludac. PGR and LERm in the progeny showed a distribution not significantly different from a normal distribution (*p* > 0.05) which was not the case for HD and summer dormancy, which showed a higher number of individuals with phenotypic values close to those of Medly (**Figures 1** and **2**). No significant maternal effect (*p* > 0.05) was found for all traits except a slight effect for summer dormancy (*p* < 0.03).

**Table 1:** Basic statistics and broad-sense heritability (H^2^) for plant growth rate (PGR), heading date (HD), summer dormancy (Dorm), and maximum leaf elongation rate (LERm) on the genotype means of the progeny, the parents (Kasbah summer dormant and Medly summer active) and a temperate genotype from the Ludac variety.

Traits	Kasbah	Medly	Ludac	Mean ± SEM^∗^	Range	H^2^
PGR (mm°C^-1^d^-1^)	0.41	0.57	0.64	0.62 ± (0.01)	0.28-1.05	0.48
HD (days)	83	76	88	75 ± 0.47	62-92	0.67
Dorm (%)	87	38	10	47 ± 1.6	6-94	0.56
LERm (mm°C^-1^d^-1^)	1.10	1.45	1.49	1.46 ± (0.10)	0.85-2.28	0.61


*Standart error of the mean.

### Correlations between Traits

**FIGURE 3:**
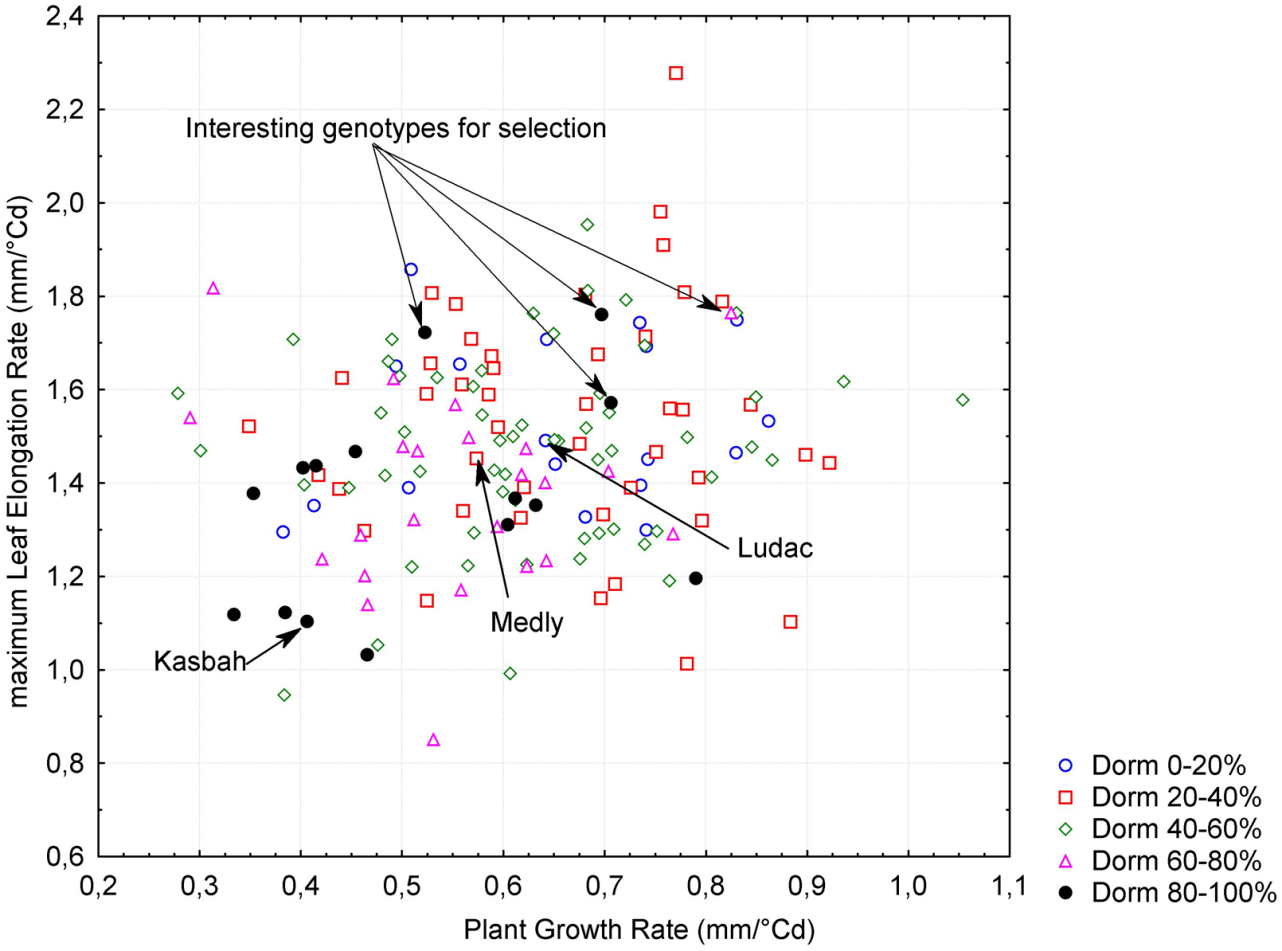
**Relationship between plant growth rate (PGR), maximum leaf elongation rate (LERm) and summer dormancy (Dorm) within the progeny between two individuals from the summer dormant cultivar Kasbah and the summer active cultivar Medly.** Interesting individuals for breeding purpose with a high level of summer dormancy and a high vegetative growth rate are indicated.

A significant negative correlation (*r* = -0.62, *p* < 0.005) was observed between PGR in spring and HD, both measured in Morocco. This is consistent with the phenotypes of the parents, with Kasbah showing a lower PGR and a later HD than Medly. PGR in spring in Morocco was positively correlated (*r* = 0.18, *p* < 0.05) with LERm in autumn in France. Summer dormancy showed a negative correlation with both PGR in spring in Morocco (*r* = -0.34, *p* < 0.005) and LERm in autumn in France (*r* = -0.27, *p* < 0.005). Nevertheless, interesting genotypes with a high level of summer dormancy and a high level of vegetative growth rate in both spring and autumn could be identified in the progeny (**Figure 3**).

### QTL Identification

One genetic map for each parent was built (**Supplementary Figures S1**–**S14**). The total length of the maps were 377 and 423 cM for Kasbah and Medly, respectively. The average distance between markers were 2.6 and 2 cM for Kasbah and Medly, respectively, but the coverage was uneven between LGs (**Table 2**). In particular, the LGs 1 and 7 of Kasbah included only two and four markers, respectively.

**Table 2:** Number of mapped markers and linkage group (LG) size for both parents of the progeny (Kasbah and Medly).

	LG1	LG2	LG3	LG4	LG5	LG6	LG7	Total
*Kasbah parent*								
Number of markers	2	38	32	16	29	26	4	147
Linkage group size (cM)	0.03	87	30	44	89	94	33	377
*Medly parent*								
Number of markers	34	20	28	40	33	29	26	210
Linkage group size (cM)	66	38	68	82	34	70	65	423


**FIGURE 4:**
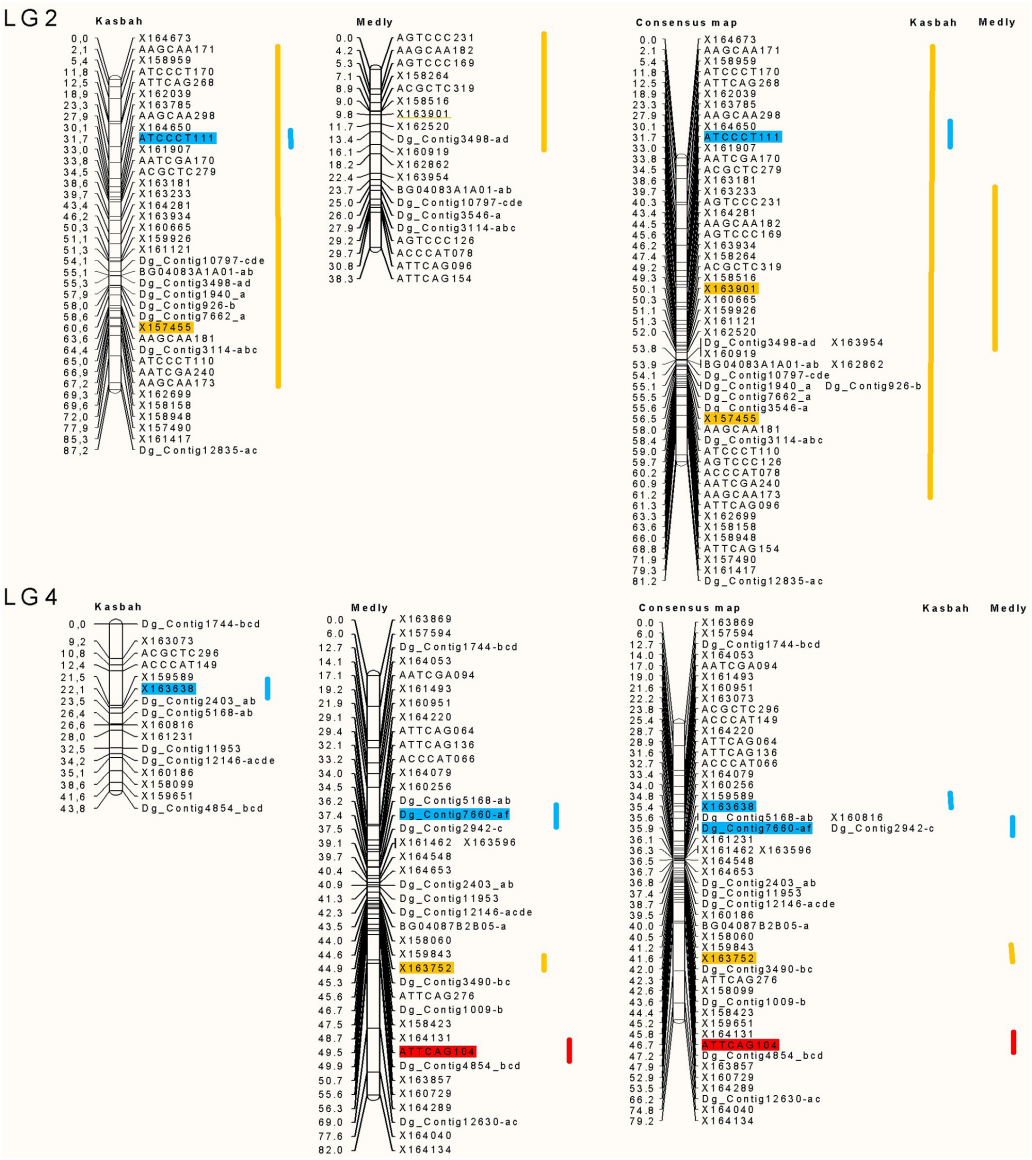
**Location of the QTL on linkage groups (LG) two and four for PGR in green (PGR in mm°C^-1^d^-1^), HD in orange (HD in days), summer dormancy in red (Dorm in %) and maximum leaf elongation rate in blue (LERm in mm°C^-1^d^-1^).** The QTL identified on each parent (Medly and Kasbah) are presented on the map of each parent separately and on a consensus map. The length of the line showing QTL location covers all significant markers (*p* < 0.01) and the markers retained by the stepwise regression are colored. The names of SSR markers start with DG_contig or BD. The names of DArT markers start with X. The names of AFLP markers consist of the three selective bases of the EcoRI primer followed by the three selective bases of the MseI primer followed by the size of the scored band, e.g., ATTCAG082.

**FIGURE 5:**
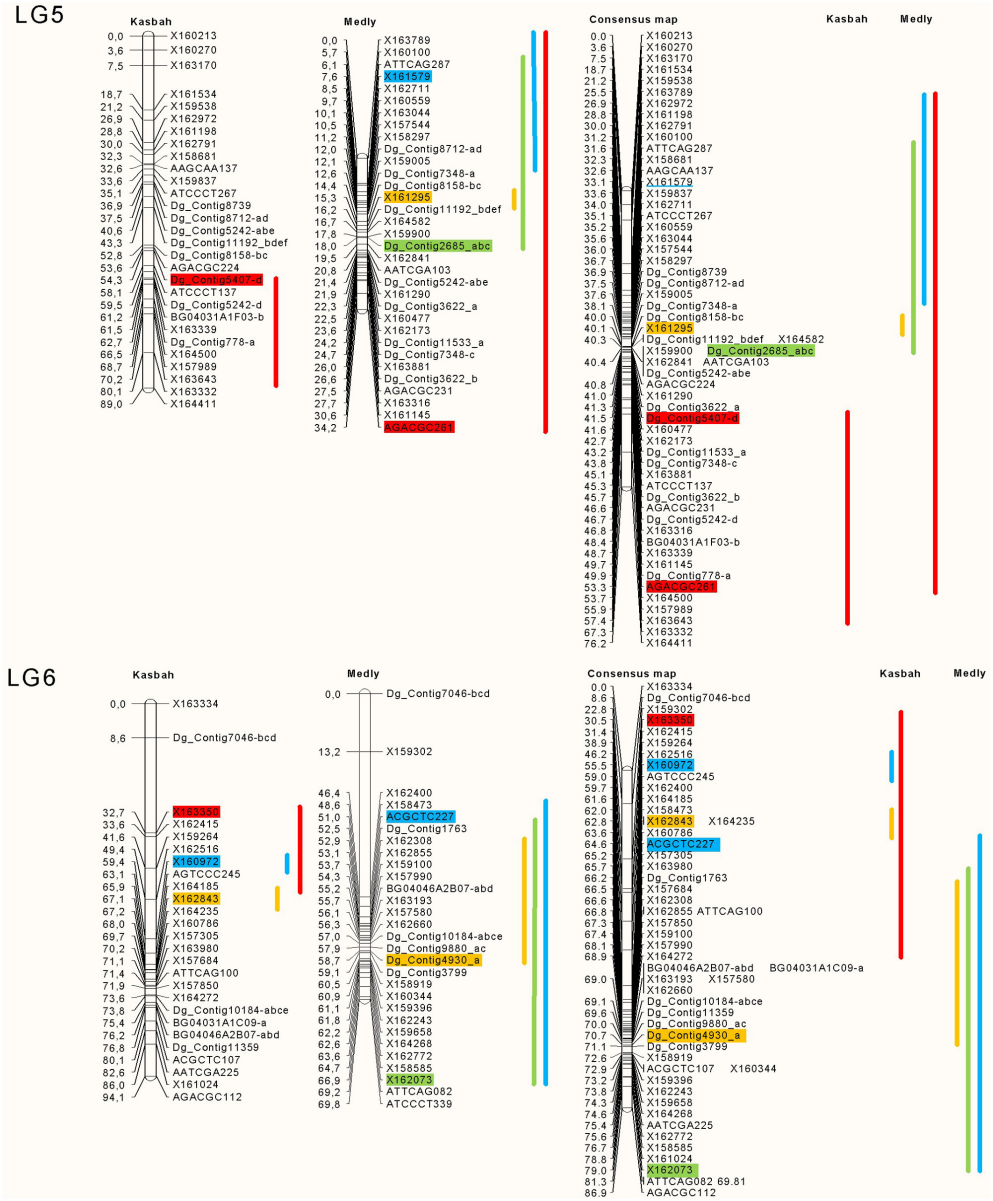
**Location of the QTL on LGs five and six for plant growth rate in green (PGR in mm°C^-1^d^-1^), HD in orange (HD in days), summer dormancy in red (Dorm in %) and maximum leaf elongation rate in blue (LERm in mm°C^-1^d^-1^).** The QTL identified on each parent (Medly and Kasbah) are presented on the map of each parent separately and on a consensus map. The length of the line showing QTL location covers all significant markers (*p* < 0.01) and the markers retained by the stepwise regression are colored. The names of SSR markers start with DG_contig or BD. The names of DArT markers start with X. The names of AFLP markers consist of the three selective bases of the EcoRI primer followed by the three selective bases of the MseI primer followed by the size of the scored band, e.g., ATTCAG082.

The position and effects of the QTL for both parents are presented in **Table 3** and **Figures 4** and **5.** For all traits except summer dormancy, the percentage of variance explained by the QTL was larger for Medly than for Kasbah. It reached 31% for HD on Medly with one major QTL on LG2. No significant QTL was detected for PGR on Kasbah. QTL for summer dormancy were detected in Kasbah, the summer dormant parent but also in Medly, the summer active parent and a common QTL was identified on LG5 in both parents. In Medly, this QTL co-localized with QTL for PGR, LERm, and HD. In Kasbah, the QTL for summer dormancy on LG6 co-localized with a QTL for LERm and HD.

**Table 3:** Quantitative trait loci (QTL) identification and effects for plant growth rate (PGR in mm°C^-1^d^-1^), heading date (HD in days), summer dormancy (Dorm in %), and maximum leaf elongation rate (LERm in mm°C^-1^d^-1^) on both parents (M: Medly and K: Kasbah).

Trait	Parent	Marker	LG	Position (cM)	Partial *R*^2^	*R*^2^ tot	Allelic effect^∗^
PGR	M	Dg_Contig2685_a	LG5	18	0.04		-0.08
	M	X162073	LG6	67	0.09	0.13	-0.11
Dorm	K	Dg_Contig5407-d	LG5	54	0.07		12.3
	K	X163350	LG6	33	0.06	0.13	-10.8
Dorm	M	ATTCAG104	LG4	32	0.07		9.7
	M	AGACGC261	LG5	34	0.05	0.12	11.5
HD	K	X157455	LG2	61	0.11		-4.2
	K	X162843	LG6	67	0.03	0.14	-3.5
HD	M	X163901	LG2	28	0.19		4.9
	M	X163752	LG4	37	0.03		-4.1
	M	X161295	LG5	15	0.01		3.4
	M	Dg_Contig4930_a	LG6	59	0.08	0.31	-3.3
LERm	K	ATCCCT111	LG2	32	0.07		-0.12
	K	X163638mp	LG4	22	0.04		-0.11
	K	X160972mp	LG6	59	0.04	0.15	-0.12
LERm	M	Dg_Contig7660-f	LG4	45	0.09		0.18
	M	X161579mp	LG5	8	0.08		0.11
	M	ACGCTC227	LG6	51	0.08	0.25	-0.12


*Effect of the presence of the allele.

## Discussion

The offspring obtained by crossing a summer dormant genotype from the Kasbah variety and a summer active genotype from the Medly variety exhibited a large quantitative variation for summer dormancy scored as the percentage of aerial senescence under summer irrigation. This result reveals that summer dormancy was not fixed in the Kasbah parent and that this trait is not controlled by one locus only but by several loci. This is in agreement with the gradual range of summer dormancy observed on Moroccan or Sicilian cocksfoot ecotypes (Shaimi et al., 2009a; Copani et al., 2012). Summer dormancy in the offspring showed transgressive segregation indicating that alleles conferring summer dormancy should be present in Medly, the summer active parent and that alleles conferring summer activity should be present in Kasbah, the summer dormant parent. A transgressive segregation has also been observed in a F1 population of tall fescue between a summer active and a summer dormant parent (Malay et al., 2011). The observed graduation of summer dormancy opens up opportunities to create varieties with different levels of summer dormancy which could be chosen depending on the targeted environment.

Plant growth rate in spring in Morocco and LERm in autumn in France were higher for Medly (summer active Mediterranean variety) than for Kasbah (summer dormant Mediterranean variety) and higher for Ludac (summer active temperate variety) than for Medly. Such a classification has been observed for dry matter yield on sward in Morocco for the two first years of harvest (Shaimi et al., 2009a). In this study, in the third year, the summer dormant variety showed a higher yield than the others due to their lack of persistency under severe droughts. Similar results have been obtained in southern Australia (Norton, 2014). Some offspring had a higher PGR and LERm than the temperate cultivar (Ludac), suggesting interesting productivity potential for forage production.

A negative correlation was observed between summer dormancy and both PGR in spring in Morocco and LERm in autumn in France. This negative correlation between summer dormancy and traits related to vegetative productivity has already been observed on a set of cocksfoot ecotypes (Shaimi et al., 2009a). Nevertheless the negative correlation observed in the present study was moderate and some genotypes with a high level of summer dormancy and a good level of PGR or LERm were observed. This result, observed after only one crossing generation, showed that the correlation among natural populations may be ascribed to an environmental adaptation of traits rather than a genetic control of the traits by the same genes or tightly linked genes. These results confirm previous preliminary observations on mini-sward sown with progenies from polycrosses between six plants of the two Kasbah and Medly varieties which had intermediate biomass production and a greater range of summer dormancy than their parents (Shaimi et al., 2009a). If this pattern is consistent in subsequent generations of crossing and in different plant material, this opens new opportunities to breed for summer dormant Mediterranean varieties with a good level of annual forage productivity by crossing temperate elite material with highly drought adapted summer dormant ecotypes. A similar strategy has been proposed by Hopkins and Bhamidimarri (2009) who noted barriers to fertility for tall fescue and cocksfoot. In the case of cocksfoot, we did not observe these barriers, which could be due to the fact that both summer active and summer dormant parents were tetraploid material.

Two genetic maps were built with a total of 325 markers. The sizes of the maps were 377 and 423 cM for the Kasbah parent and the Medly parent, respectively. These sizes were relatively small compared to those of Song et al. (2011): 562 and 745 cM or those of Xie et al. (2012a): 947 and 1038 cM. A part of the explanation could originate from the use of the “simulated annealing ordering” option of TetraploidMap which has a tendency to shrink the LG length. Moreover, this lack of coverage could be due to a segregation distortion and/or to a lack of polymorphic markers. In the future, the number of markers could be greatly increased by using genotyping by sequencing (GBS; Elshire et al., 2011) in addition to some codominant markers with several alleles as SSR. Another advantage of GBS markers besides their low cost is the possibility to estimate the allele dosage in polyploidy species (Garcia et al., 2013) and to take into account this dosage in map construction and QTL detection (Hackett et al., 2013, 2014). Nevertheless, all homologous chromosomes for all LGs were well-represented with the exception of LG1 and 7 for the Kasbah parent.

Our study is the first to propose QTL identification for summer dormancy in cocksfoot and is the second study on QTL identification in this complex auto-tetraploid species (Xie et al., 2012a). QTL identification in both parents revealed that at least three different genomic regions were involved in summer dormancy variation (LG 4, 5, and 6). The QTL on LG4 was found only in the parent Medly and the QTL on LG6 was found only in the parent Kasbah. This result shows that different genes in both parents explain the variation of summer dormancy observed in the offspring. The QTL on LG5 was found in both parents and the locations on the consensus map suggest that this QTL should be the same in both parents. It would be interesting to increase the accuracy of the map in this region in order to identify the gene under this common QTL.

Some genomic regions were specifically involved in either dormancy or PGR or LERm. This explains why dormancy and growth were not highly correlated. Conversely, some co-locations were identified between summer dormancy and PGR or LERm, which could contribute to the negative correlation between these traits. For each of these regions, fine mapping should be performed in order to analyze further whether one gene with pleiotropic effects or several linked genes are involved in the variation of the traits. For these regions, if several genes are involved, it would also be interesting for a breeding purpose to identify the phase, i.e., coupling *versus* repulsion, between alleles for summer dormancy and productivity traits.

Heading date QTL were identified on LG2 and 6 for both parents and on LG4 and 5 for the parent Medly. The QTL on LG2 was the major one for both parents explaining 11 and 19% of the phenotypic variance for the Kasbah and Medly parents, respectively. The locations of this QTL for both parents on the consensus map show that it should be the same QTL for both parents. In a former study (Xie et al., 2012a) on a “F1 population” between a very late heading *D. glomerata* subsp. *himalayensis* parent and an early to mid-heading *D. glomerata* subsp. *aschersoniana* parent, HD QTL have been identified on LG2 and 6 for both parents and LG 5 for the *himalayensis* parent. On LG2 the QTL were close to the markers Contig_10797 and Contig_3498 in both studies. On LG6 the QTL were close to the marker Contig_10184 in both studies. On LG5 the QTL were close to the markers Contig_8712 and Contig_5242 in both studies. This coincidence suggests that a few major genes explain variation for HD in *Dactylis* sp. HD QTL have been identified on all the seven LGs in perennial ryegrass (*Lolium perenne* L.; Shinozuka et al., 2012). Fine mapping and synteny could be used to identify candidate genes underpinning QTL.

## Conclusion

The use of summer dormancy in forage perennial grasses to improve their survival under severe drought has been previously considered (Lelievre et al., 2008; Clark and Harris, 2009; Culvenor, 2009; Hopkins and Bhamidimarri, 2009). Nevertheless summer dormancy has often been reported as negatively correlated to annual forage production (Shaimi et al., 2009a). The present study demonstrates that at least a part of this negative correlation could be broken by crossing summer dormant with summer active and highly productive genotypes. A first genetic dissection of these traits shows that more than one locus was involved in summer dormancy variability and that limited co-locations between QTL for summer dormancy and production traits exist. These results open the way for breeding strategies involving different genetic resources adapted to Mediterranean climates crossed with elite temperate plant materials in order to create pre-breeding pools. Molecular markers will be helpful to construct new productive Mediterranean varieties. Drought resilience of perennial forage species combined to high biomass productivity remains a breeding priority for grass species used in animal farming systems in rain-fed areas under climate change.

## Author Contributions

RK was the project leader for Morocco and contributed to the phenotyping in Morocco, to data analyses and to writing the manuscript.

LZ contributed to the phenotyping in Morocco, to AFLP analyses and data analyses.

FV contributed to the setting up of the project, to the phenotyping of summer dormancy, to data analyses and to writing the manuscript.

AG performed the analysis of raw DArT data and the construction of the genetic maps.

BJ contributed to the setting up of the project, to the construction of the genetic maps and to writing the manuscript.

NS contributed to the phenotyping in Morocco and to AFLP analyses.

MF contributed to collecting phenotypic data in Morroco.

PB was the project leader for France and contributed to data analyses and to writing the manuscript.

## Conflict of Interest Statement

The authors declare that the research was conducted in the absence of any commercial or financial relationships that could be construed as a potential conflict of interest.

## References

[B1] Annicchiarico P. Pecetti L. Bouzerzour H. Kallida R. Khedim A. Porqueddu C. (2011). Adaptation of contrasting cocksfoot plant types to agricultural environments across the Mediterranean basin. *Environ. Exp. Bot.* 74 82–89. 10.1016/j.envexpbot.2011.05.002

[B2] Auzanneau J. Huyghe C. Escobar-Gutiérrez A. J. Julier B. Gastal F. Barre P. (2011). Association study between the gibberellic acid insensitive gene and leaf length in a *Lolium perenne* L. synthetic variety. *BMC Plant Biol.* 11:183 10.1186/1471-2229-11-183 PMC3292539 22204490

[B3] Bushman B. S. Larson S. R. Tuna M. West M. S. Hernandez A. G. Vullaganti D. (2011). Orchardgrass (*Dactylis glomerata* L.) EST and SSR marker development, annotation, and transferability. *Theor. Appl. Genet.* 123 119–129. 10.1007/s00122-011-1571-2 21465186

[B4] Clark S. Harris C. (2009). Summer dormancy in Australian perennial grasses: historical background, a simulation study, and current research. *Crop Sci.* 49 2328–2334. 10.2135/cropsci2009.06.0322

[B5] Copani V. Testa G. Lombardo A. Cosentino S. L. (2012). Evaluation of populations of *Dactylis glomerata* L. native to Mediterranean environments. *Crop Pasture Sci.* 63 1124–1134. 10.1071/cp12276

[B6] Culvenor R. A. (2009). Breeding and use of summer-dormant grasses in Southern Australia, with special reference to phalaris. *Crop Sci.* 49 2335–2346. 10.2135/cropsci2009.06.0321

[B7] Culvenor R. A. Boschma S. P. (2005). Evaluation of phalaris (*Phalaris aquatica* L.) germplasm for persistence under grazing on the North-West Slopes, New South Wales. *Aust. J. Agric. Res.* 56 731–741. 10.1071/ar04300

[B8] Elshire R. J. Glaubitz J. C. Sun Q. Poland J. A. Kawamoto K. Buckler E. S. (2011). A robust, simple genotyping-by-sequencing (GBS) approach for high diversity species. *PLoS ONE* 6:e19379 10.1371/journal.pone.0019379 PMC3087801 21573248

[B9] Garcia A. A. F. Mollinari M. Marconi T. G. Serang O. R. Silva R. R. Vieira M. L. C. (2013). SNP genotyping allows an in-depth characterisation of the genome of sugarcane and other complex autopolyploids. *Sci. Rep.* 3 sre03399 10.1038/srep03399 PMC3844970 24292365

[B10] Ghesquiére M. (2013). “DArTClimate: développement d’outils moléculaires dédiés à l’amélioration des complexes d’espèces fourragers en réponse au changement climatique,” in *Proceedings of the Séminaire AIP Bio-Ressources 2010-2011*, Paris, 175–185.

[B11] Ghesquière M. Barre P. Durand J. L. Julier B. Litrico I. Maamouri A. (2012). “Construction of a DArT marker ressource for better adapted forage crops to climate change,” in *Proceedings of the 7th International Symposium on the Molecular Breeding of Forage and Turf, MBFT2012 2012 June 4–7*, eds Bushman B. Spangenberg G. C. (Salt Lake City, UT), 85.

[B12] Hackett C. A. Bradshaw J. E. Bryan G. J. (2014). QTL mapping in autotetraploids using SNP dosage information. *Theor. Appl. Genet.* 127 1885–1904. 10.1007/s00122-014-2347-2 24981609 PMC4145212

[B13] Hackett C. A. Bradshaw J. E. Meyer R. C. McNicol J. W. Milbourne D. Waugh R. (1998). Linkage analysis in tetraploid species: a simulation study. *Genet. Res.* 71 143–154. 10.1017/S0016672398003188

[B14] Hackett C. A. Luo Z. W. (2003). TetraploidMap: construction of a linkage map in autotetraploid species. *J. Hered.* 94 358–359. 10.1093/jhered/esg066 12920109

[B15] Hackett C. A. McLean K. Bryan G. J. (2013). Linkage analysis and QTL mapping using SNP dosage data in a tetraploid potato mapping population. *PLoS ONE* 8:e63939 10.1371/journal.pone.0063939 PMC3660524 23704960

[B16] Hazard L. (1996). Plasticity gives a greater flexibility to forage grass use. *Fourrages* 147 293–302.

[B17] Hazard L. Ghesquière M. (1997). Productivity under contrasting cutting regimes of perennial ryegrass selected for short and long leaves. *Euphytica* 95 295–299. 10.1023/A:1003048316012

[B18] Hirata M. Yuyama N. Cai H. (2011). Isolation and characterization of simple sequence repeat markers for the tetraploid forage grass *Dactylis glomerata*. *Plant Breed.* 130 503–506. 10.1111/j.1439-0523.2010.01831.x

[B19] Hopkins A. A. Bhamidimarri S. (2009). Breeding summer-dormant grasses for the United States. *Crop Sci.* 49 2359–2362. 10.2135/cropsci2009.06.0329

[B20] Horst G. L. Nelson C. J. Asay K. H. (1978). Relationship of leaf elongation to forage yield of tall fescue genotypes. *Crop Sci.* 18 715–719. 10.2135/cropsci1978.0011183X001800050005x

[B21] IPCC (2014). *Contribution of Working Groups I, II and III to the Fifth Assessment Report of the Intergovernmental Panel on Climate Change.* Climate Change 2014: Synthesis Report Geneva: IPCC.

[B22] Lelievre F. Norton M. R. Volaire F. (2008). “Perennial grasses in rainfed Mediterranean farming systems – current and potential role,” in *Sustainable Mediterranean Grasslands and Their Multi-Functions*, eds Porqueddu C. Tavares de Sousa M. M. (Zaragoza: CIHEAM/FAO/ENMP/SPPF), 13 7–146.

[B23] Lelievre F. Seddaiu G. Ledda L. Porqueddu C. Volaire F. (2011). Water use efficiency and drought survival in Mediterranean perennial forage grasses. *Field Crops Res.* 121 333–342. 10.1016/j.fcr.2010.12.023

[B24] Lelievre F. Volaire F. (2009). Current and potential development of perennial grasses in rainfed mediterranean farming systems. *Crop Sci.* 49 2371–2378. 10.2135/cropsci2009.06.0324

[B25] Malay S. Suresh B. Azhaguvel P. Andrew H. (2011). “Deciphering summer dormancy in tall fescue,” in *Proceedings of the ASA-CSSA-SSSA International Annual Meetings; 2011 Oct 16-19*, San Antonio, TX, 235–239.

[B26] Malinowski D. P. Pinchak W. E. (2015). Summer dormancy trait as a strategy to provide perennial cool-season grass forage alternatives in southern latitude environments affected by climate change. *Agron. J.* 107 1227–1234. 10.2134/agronj14.0628

[B27] Nie Z. N. Chapman D. F. Tharmaraj J. Clements R. (2004). Effects of pasture species mixture, management, and environment on the productivity and persistence of dairy pastures in south-west Victoria. 1. Herbage accumulation and seasonal growth pattern. *Aust. J. Agric. Res.* 55 625–636. 10.1071/ar03174

[B28] Nie Z. N. Miller S. Moore G. A. Hackney B. F. Boschma S. P. Reed K. F. M. (2008). Field evaluation of perennial grasses and herbs in southern Australia. 2. Persistence, root characteristics and summer activity. *Aust. J. Exp. Agric.* 48 424–435. 10.1071/ea07136

[B29] Norton G. M. (2014). *Summer Dormant Cocksfoot A Great Pasture Option.* Perth, WA: Farming Ahead, 266.

[B30] Norton M. R. Lelievre F. Fukai S. Volaire F. (2008). Measurement of summer dormancy in temperate perennial pasture grasses. *Aust. J. Agric. Res.* 59 498–509. 10.1071/ar07343

[B31] Norton M. R. Lelievre F. Volaire F. (2006a). Summer dormancy in *Dactylis glomerata* L.: the influence of season of sowing and a simulated mid-summer storm on two contrasting cultivars. *Aust. J. Agric. Res.* 57 565–575. 10.1071/ar05237

[B32] Norton M. R. Volaire F. Lelievre F. (2006b). Summer dormancy in *Festuca arundinacea* Schreb.; the influence of season of sowing and a simulated mid-summer storm on two contrasting cultivars. *Aust. J. Agric. Res.* 57 1267–1277. 10.1071/ar06082

[B33] Oram R. N. (1990). *Register of Australian Herbage Plant Cultivars*, 3rd Edn Melbourne, VIC: CSIRO Publications.

[B34] Pauly L. Flajoulot S. Garon J. Julier B. Beguier V. Barre P. (2012). Detection of favorable alleles for plant height and crown rust tolerance in three connected populations of perennial ryegrass (*Lolium perenne* L.). *Theor. Appl. Genet.* 124 1139–1153. 10.1007/s00122-011-1775-5 22234605

[B35] Pecetti L. Annicchiarico P. Abdelguerfi A. Kallida R. Mefti M. Porqueddu C. (2011). Response of Mediterranean tall fescue cultivars to contrasting agricultural environments and implications for selection. *J. Agron. Crop Sci.* 197 12–20. 10.1111/j.1439-037X.2010.00443.x

[B36] Phung N. T. Mai C. D. Mournet P. Frouin J. Droc G. Ta N. K. (2014). Characterization of a panel of Vietnamese rice varieties using DArT and SNP markers for association mapping purposes. *BMC Plant Biol.* 14:371 10.1186/s12870-014-0371-7 PMC4279583 25524444

[B37] Reed K. F. M. Smith K. F. Jahufer Z. Anderson M. Caradus J. (1999). “Alleviating the impact of summer moisture stress in perennial pasture,” in *Proceedings of the 11th Australian Plant Breeding Conference 1999 19–23 April, Adelaide, SA*, ed. Langridgep P. (Glen Osmond, SA: CRC for Molecular Plant Breeding), 50.

[B38] Reeder L. Sleper D. Nelson C. (1984). Response to selection for leaf area expansion rate of tall fescue. *Crop Sci.* 24 97–100. 10.2135/cropsci1984.0011183X002400010022x

[B39] Rhodes I. (1969). The relationship between productivity and some components of canopy structure in ryegrass (*Lolium* spp.). I. Leaf length. *J. Agric. Sci.* 73 315–319. 10.1017/S0021859600019924

[B40] Rhodes I. (1971). The relationship between productivity and some components of canopy structure in ryegrass (*Lolium* spp.) II. Yield, canopy structure and ligth interception. *J. Agric. Sci.* 77 283–292. 10.1017/S0021859600024436

[B41] Rhodes I. (1973). The relationship between productivity and some components of canopy structure in ryegrass (*Lolium* spp.) III. Spaced plant characters, their heritabilities and relationship to sward yield. *J. Agric. Sci.* 80 171–176. 10.1017/S002185960005718X

[B42] Rhodes I. Mee S. (1980). Changes in dry matter yield associated with selection for canopy characters in ryegrass. *Grass Forage Sci.* 35 35–39. 10.1111/j.1365-2494.1980.tb01490.x

[B43] SAS Institute (2011). *The SAS System for Windows. Release 9.2.* Cary, NC: SAS Institute Inc.

[B44] Shaimi N. Kallida R. Volaire F. Al Faiz C. (2009a). Summer dormancy in orchardgrass: evaluation and characterization through ecophysiological and genetic studies. *Crop Sci.* 49 2353–2358. 10.2135/cropsci2009.06.0325

[B45] Shaimi N. Kallida R. Volaire F. Saidi N. Al Faiz C. (2009b). Summer dormancy and drought survival of Moroccan ecotypes of orchardgrass. *Crop Sci.* 49 1416–1424. 10.2135/cropsci2008.09.0545

[B46] Shinozuka H. Cogan N. O. I. Spangenberg G. C. Forster J. W. (2012). Quantitative Trait Locus (QTL) meta-analysis and comparative genomics for candidate gene prediction in perennial ryegrass (*Lolium perenne* L.). *BMC Genet.* 13:101 10.1186/1471-2156-13-101 PMC3532372 23137269

[B47] Sleper D. A. West C. P. (1996). “Tall fescue,” in *Cool Season Forage Grasses*, eds Moser D. R. B. Casler M. D. (Madison, WI: Agronomy Monograph 34. ASA), 471–502.

[B48] Song Y. Liu F. Zhu Z. Tan L. Fu Y. Sun C. (2011). Construction of a simple sequence repeat marker-based genetic linkage map in the autotetraploid forage grass *Dactylis glomerata* L. *Grassl. Sci.* 57 158–167. 10.1111/j.1744-697X.2011.00223.x

[B49] Sosnowski O. De Oliveira Y. Moreau L. Charcosset A. Joets J. Jorge V. (2012). *BioMercator. Un Logiciel Dédié à la Compilation de Cartes Génétiques et la Meta-Analyse de QTLs [Application].* Available at: http://prodinra.inra.fr/record/255679

[B50] StatSoft (2003). *STATISTICA Pour Windows. StatSoft France.* Available at: http://www.statsoft.fr

[B51] Volaire F. (2002). Drought survival, summer dormancy and dehydrin accumulation in contrasting cultivars of *Dactylis glomerata*. *Physiol. Plant.* 116 42–51. 10.1034/j.1399-3054.2002.1160106.x 12207661

[B52] Volaire F. Barkaoui K. Norton M. (2014). Designing resilient and sustainable grasslands for a drier future: adaptive strategies, functional traits and biotic interactions. *Eur. J. Agron.* 52 81–89. 10.1016/j.eja.2013.10.002

[B53] Volaire F. Barre P. Beguier V. Bourgoin T. Durand J. L. Ghesquiere M. (2013). What forage plant ideotypes for grassland that is adapted to climate change? *Fourrages* 214 119–126.

[B54] Volaire F. Norton M. (2006). Summer dormancy in perennial temperate grasses. *Ann. Bot.* 98 927–933. 10.1093/aob/mcl195 17028299 PMC2803600

[B55] Vos P. Hogers R. Bleeker M. Reijans M. Vandelee T. Hornes M. (1995). AFLP: a new technique for DNA fingerprinting. *Nucleic Acids Res.* 23 4407–4414. 10.1093/nar/23.21.4407 7501463 PMC307397

[B56] Xie W. Robins J. G. Bushman B. S. (2012a). A genetic linkage map of tetraploid orchardgrass (*Dactylis glomerata* L.) and quantitative trait loci for heading date. *Genome* 55 360–369. 10.1139/g2012-026 22551303

[B57] Xie W. Zhang X. Cai H. Huang L. Peng Y. Ma X. (2012b). Genetic maps of SSR and SRAP markers in diploid orchardgrass (*Dactylis glomerata* L.) using the pseudo-testcross strategy. *Genome* 54 212–221. 10.1139/g10-111 21423284

