# Supplementary material for: Combining Drought Survival via Summer Dormancy and Annual Biomass Productivity in Dactylis glomerata L
Source: Front Plant Sci. 2016 Feb 9;7:82. doi: 10.3389/fpls.2016.00082 (PMC4746912; doi:10.3389/fpls.2016.00082)
Supplement: Supplementary file 1 [file Presentation_1.PDF]

Supplementary Material

Combining drought survival via summer dormancy and annual biomass productivity in *Dactylis glomerata* L.

Rajae Kallida[1], Latifa Zhouri[1,2], Florence Volaire[3], Adrien Guerin[4], Bernadette Julier[4], Naima Shaimi[5], Malika Fakiri[2], Philippe Barre[4]*

*** Correspondence:** Philippe Barre, Unité de Recherche PluridisciplinairePrairies et Plantes, INRA, BAP, CS80006, Lusignan, 86600, France

philippe.barre@lusignan.inra.fr

Supplementary Figures

Genetic maps of Kasbah (Figures 1-7) and Medly (Figures 8-14) including for each linkage group the four homologous chromosomes and the combined map.

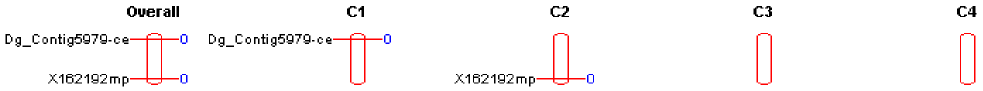

Supplementary Figure 1. Genetic maps of Kasbah linkage group 1.

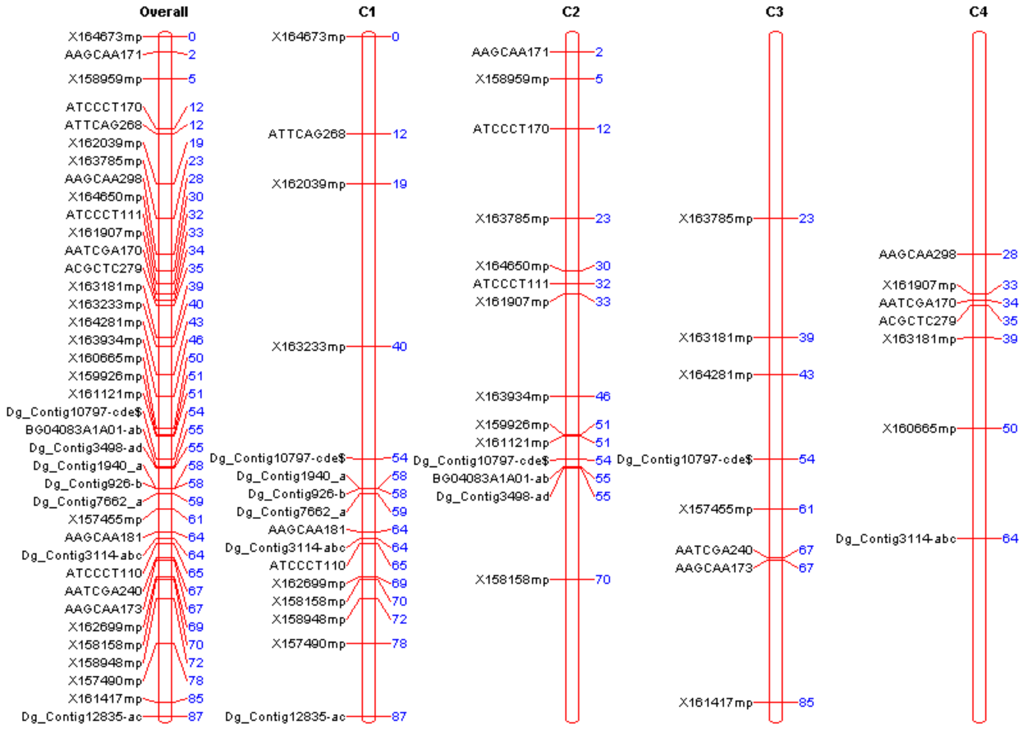

Supplementary Figure 2. Genetic maps of Kasbah linkage group 2.

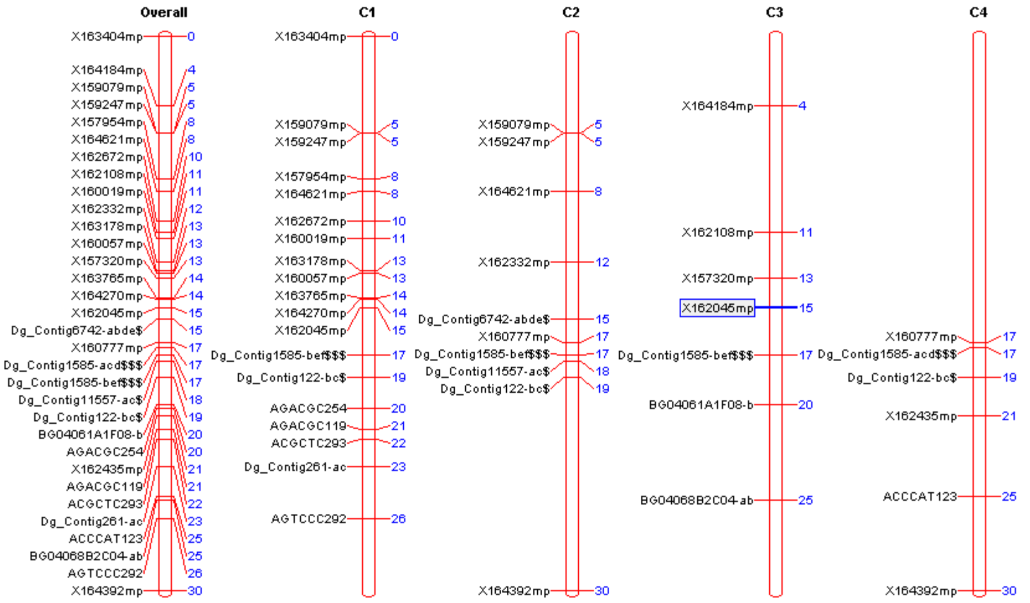

Supplementary Figure 3. Genetic maps of Kasbah linkage group 3.

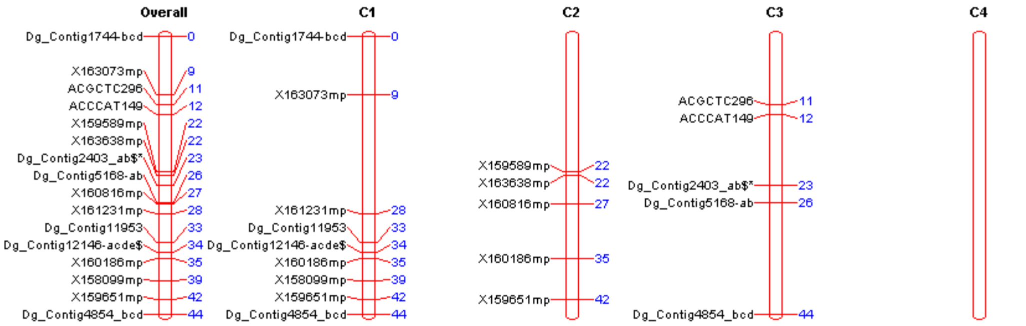

Supplementary Figure 4. Genetic maps of Kasbah linkage group 4.

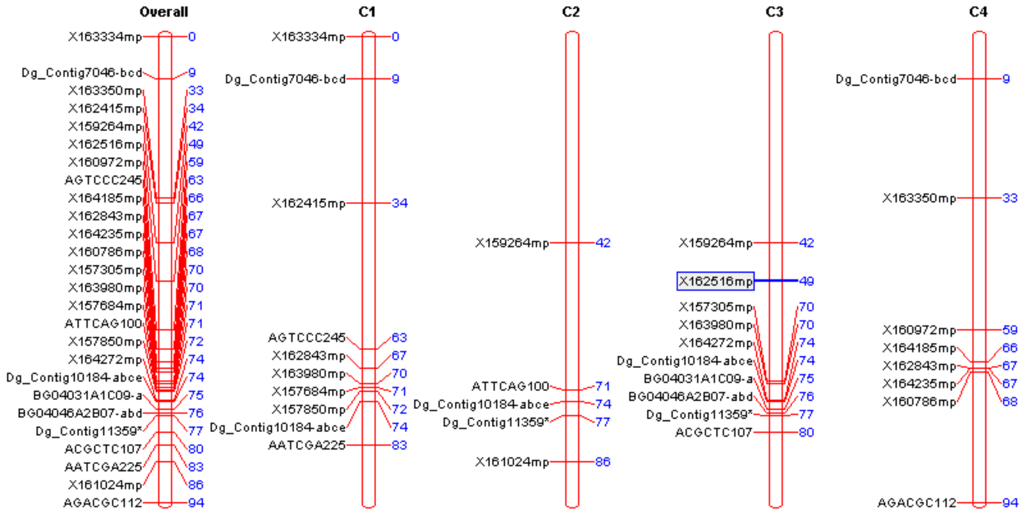

Supplementary Figure 5. Genetic maps of Kasbah linkage group 5.

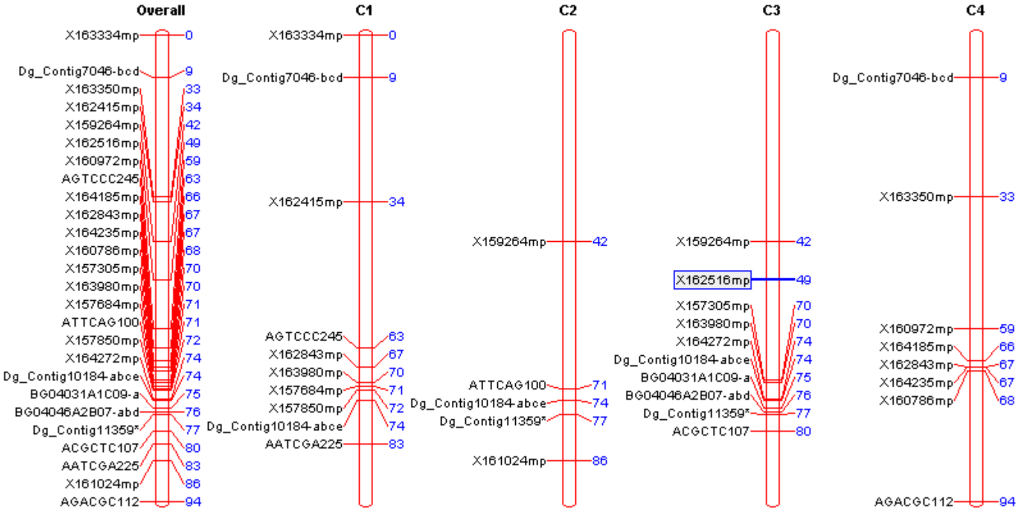

Supplementary Figure 6. Genetic maps of Kasbah linkage group 6.

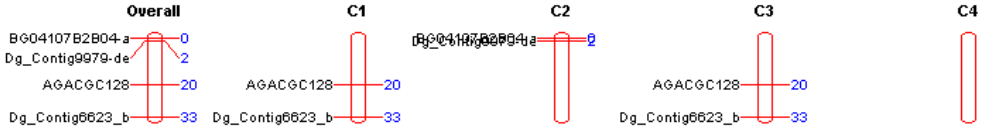

Supplementary Figure 7. Genetic maps of Kasbah linkage group 7.

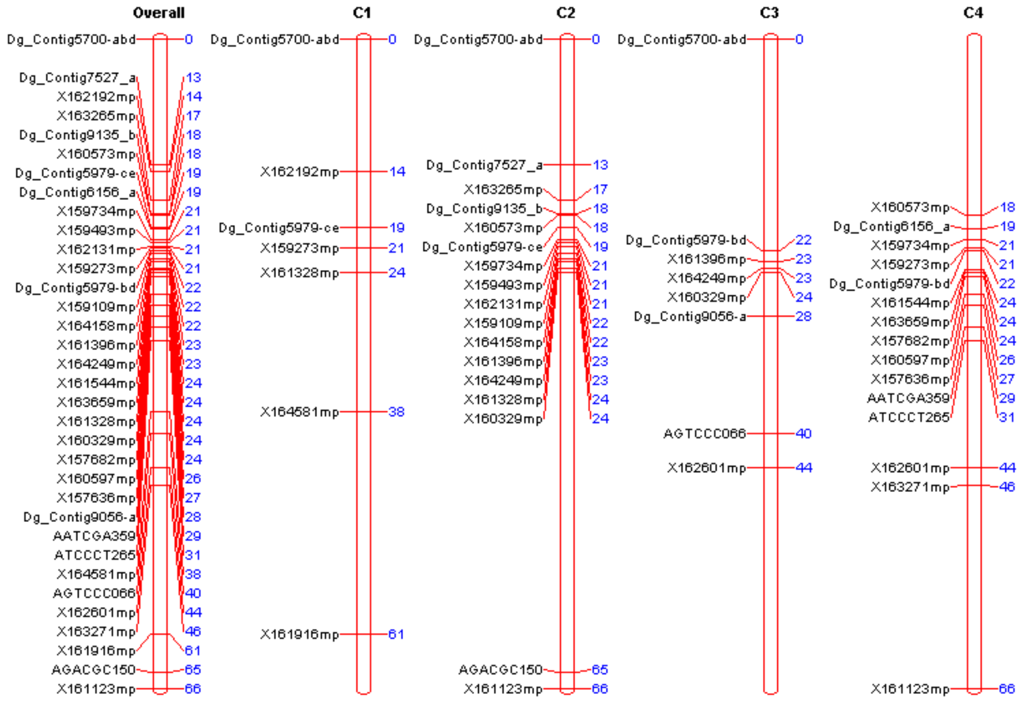

Supplementary Figure 8. Genetic maps of Medly linkage group 1.

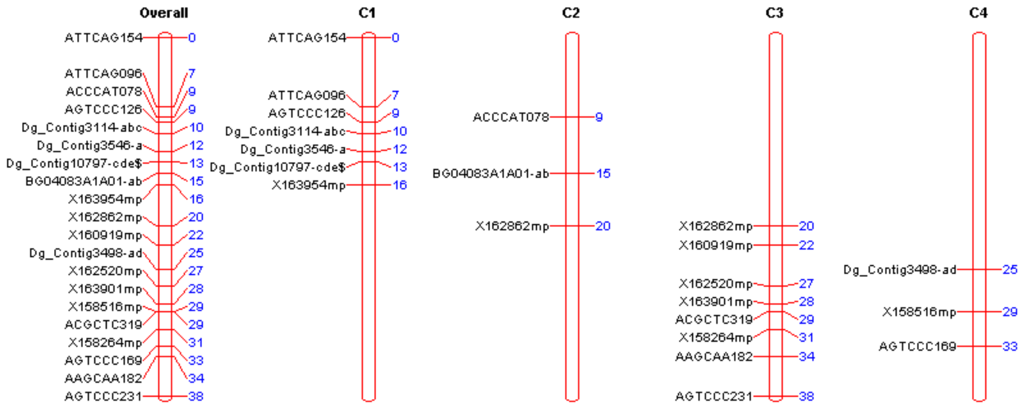

Supplementary Figure 9. Genetic maps of Medly linkage group 2.

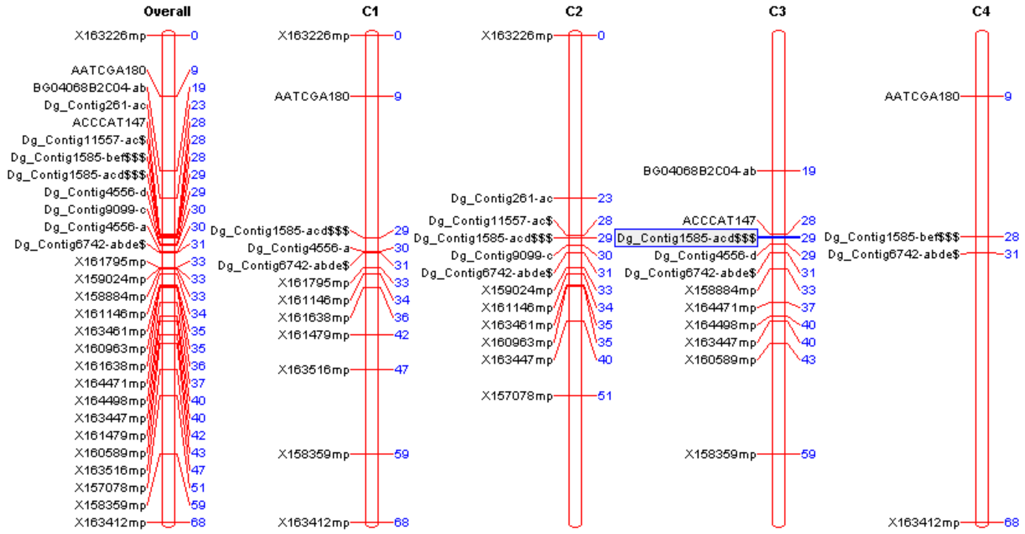

Supplementary Figure 10. Genetic maps of Medly linkage group 3.

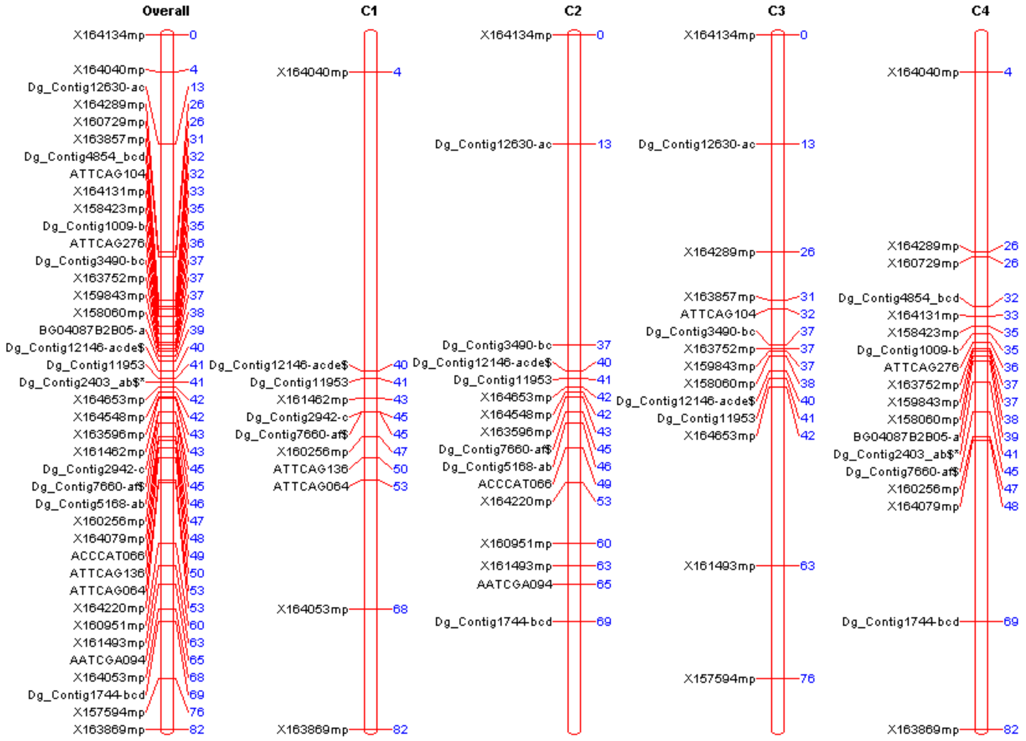

Supplementary Figure 11. Genetic maps of Medly linkage group 4.

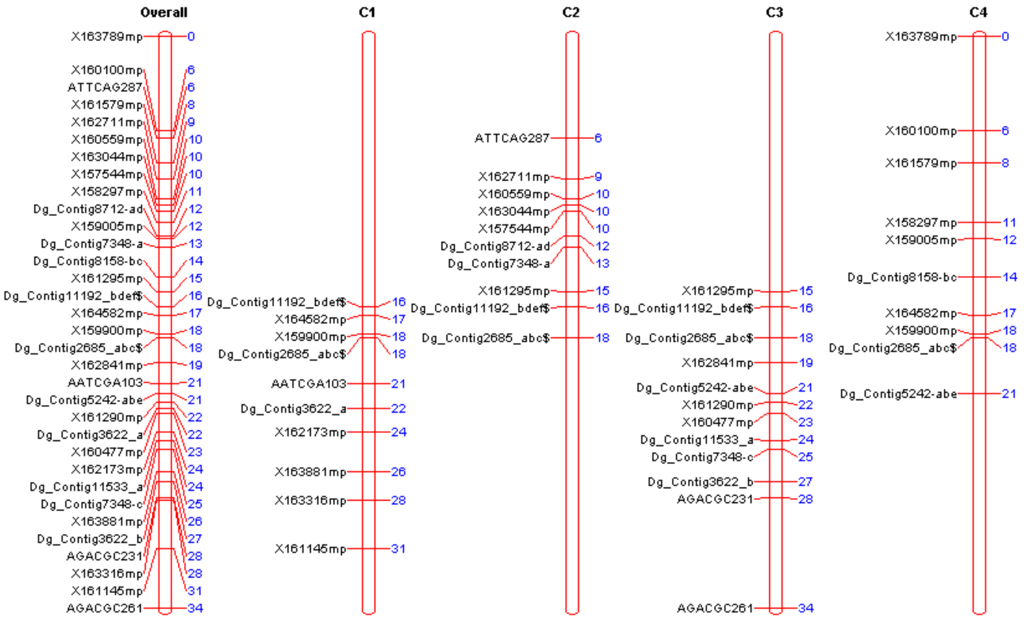

Supplementary Figure 12. Genetic maps of Medly linkage group 5.

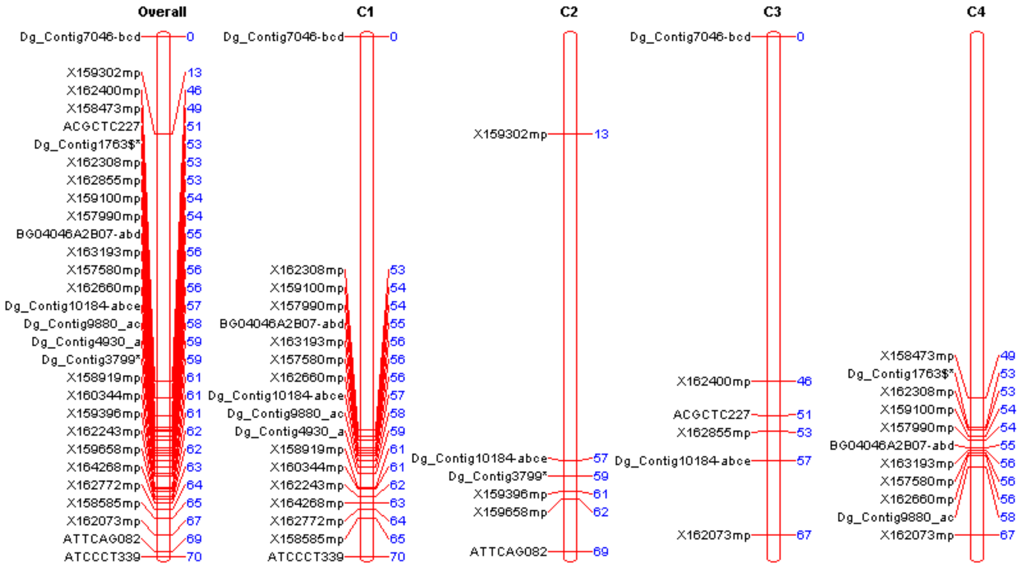

Supplementary Figure 13. Genetic maps of Medly linkage group 6.

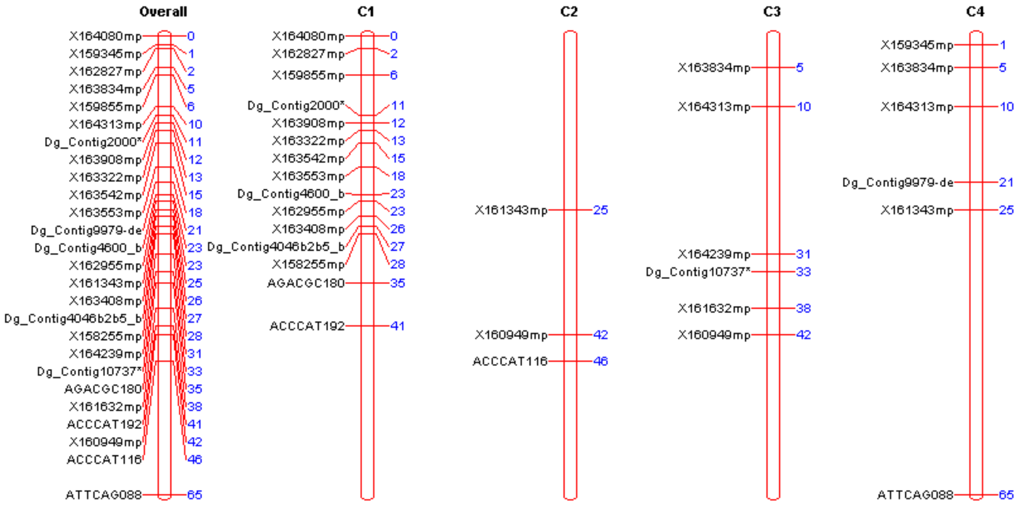

Supplementary Figure 14. Genetic maps of Medly linkage group 7.
